# *In Vitro* Selection and Characterization of New Probiotic Candidates from Table Olive Microbiota

**DOI:** 10.1371/journal.pone.0094457

**Published:** 2014-04-08

**Authors:** Cristian Botta, Tomaz Langerholc, Avrelija Cencič, Luca Cocolin

**Affiliations:** 1 Department of Forestry, Agriculture and Food Sciences, University of Torino, Torino, Italy; 2 Department of Microbiology, Biochemistry, Molecular Biology and Biotechnology, Faculty of Agriculture and Life Sciences, University of Maribor, Maribor, Slovenia; Institut Pasteur Paris, France

## Abstract

To date, only a few studies have investigated the complex microbiota of table olives in order to identify new probiotic microorganisms, even though this food matrix has been shown to be a suitable source of beneficial lactic acid bacteria (LAB). Two hundred and thirty eight LAB, belonging to *Lactobacillus plantarum*, *Lactobacillus pentosus* and *Leuconostoc mesenteroides* species, and isolated from *Nocellara Etnea* table olives, have been screened in this survey through an *in* *vitro* approach. A simulation of transit tolerance in the upper human gastrointestinal tract, together with autoaggregation and hydrophobicity, have been decisive in reducing the number of LAB to 17 promising probiotics. None of the selected strains showed intrinsic resistances towards a broad spectrum of antibiotics and were therefore accurately characterized on an undifferentiated and 3D functional model of the human intestinal tract made up of H4-1 epithelial cells. As far as the potential colonization of the intestinal tract is concerned, a high adhesion ratio was observed for *Lb. plantarum* O2T60C (over 9%) when tested in the 3D functional model, which closely mimics real intestinal conditions. The stimulation properties towards the epithelial barrier integrity and the *in* *vitro* inhibition of *L. monocytogenes* adhesion and invasion have also been assessed. *Lb. plantarum* S1T10A and S11T3E enhanced trans-epithelial electrical resistance (TEER) and therefore the integrity of the polarized epithelium in the 3D model. Moreover, S11T3E showed the ability to inhibit *L. monocytogenes* invasion in the undifferentiated epithelial model. The reduction in *L. monocytogenes* infection, together with the potential enhancement of barrier integrity and an adhesion ratio that was above the average in the 3D functional model (6.9%) would seem to suggest the *Lb. plantarum* S11T3E strain as the most interesting candidate for possible *in* *vivo* animal and human trials.

## Introduction

In the past, the gastro intestinal tract (GIT) was considered the main potential source of probiotic bacteria, but the scientific community has recently focused attention on fermented foods, recognizing them as valid and heterogeneous sources of probiotic microorganism. Although dairy products have been exploited extensively as both a source and a carrier of lactic acid bacteria (LAB) or bifidobacteria, few researches have been focused on fermented vegetable products. Their native microbiota offer a broad range of LAB species, such as *Lactobacillus* (*Lb.*) *plantarum, Lb. casei*, *Lb. paracasei*, *Lb. delbrueckii* and *Lb. brevis,* all of which present strains with probiotic features [1].

In this context, table olives are the most important fermented vegetables on the international food market, and their spontaneous fermentation, which occurs in different production processes, is usually the result of the competitive activities of the autochthonous microbiota, together with a variety of contaminating microorganisms from fermentation environments. This fermentation is mainly caused by the synergic metabolic activity of yeasts and LAB. It is generally recognized that LAB are the main inducers of brine acidification and are therefore fundamental for the stability of the final product, whereas yeasts are mainly involved in the development of the organoleptic characteristics [2]. As far as LAB are concerned, *Lb. plantarum* and *Lb. pentosus* are the most representative species involved in fermentation. The LAB microbiota of table olives is also characterized by the presence of *Lb. casei* and heterofermentative cocci, such as *Leuconostoc* (*Ln*.) *mesenteroides* [3]. All of these species have shown probiotic potentiality in many studies. For example, *in* *vitro Lb. plantarum* tests have highlighted their ability to modulate the immune response and to potentially inhibit pathogens [4], [5], as well as strains belonging to *Lb. casei* and *Lb. paracasei* species, which have proved able to inhibit Gram negative pathogens [6], [7] and *Listeria* (*L.*) *monocytogenes* [8]. *In vitro* inhibition of *L. monocytogenes* infection was recently discovered for *Ln. mesenteroides* species [9]. Table olives could also be regarded as a promising probiotic food considering that, compared to dairy products, they do not pose problems for those people who are intolerant to milk and milk products or those who need low-cholesterol diets. Moreover, it should be pointed out that an edible portion of about 100 g of olives contains more than 10^9^ live cells of selected *Lb. paracasei* or *Lb. plantarum* strains, which corresponds to the daily dose recommended to obtain beneficial effects [10].

The use of table olives as a probiotic source has already been explored in several studies [11], [12], [13], which, through *in* *vitro* methods, have evaluated the probiotic and technological characteristics of autochthonous LAB isolated from table olive fermentations. These studies have confirmed that table olives area suitable source of probiotic LAB [14]. They have also highlighted the importance of an *in* *vitro* approach as the first step towards a rational selection of new probiotics, which should take into account criteria such as antibiotic resistance and survival ability in simulated GIT conditions, and the interaction with epithelial human cells.

As established by the Food and Agriculture Organization and the World Health Organization (FAO/WHO), a potential probiotic LAB has to be Generally Recognized as Safe (GRAS) and a possible resistance towards antibiotics is the main undesirable feature [15]. Genetic resistance to antibiotics might be transferred from LAB to other commensal microorganisms through plasmids or conjugative transposons, thus increasing the danger of the pathogens that could be present in the gut environment [16]. As far as the survival ability in simulated GIT conditions is concerned, many different simulations of human digestion have been reported in literature [17] – [21]. These *in* *vitro* assessments of digestion resistance differ by the transit time, the modality of probiotic assimilation (alone or inserted in food matrices), and the complexity of the GIT model. These different simulations of the GIT conditions are extensively used as first discriminatory test for the selection of potential probiotic candidates [22].

Moreover, it is universally recognized that probiotics must be able to colonise the digestive tract [1], [23], On the other hand, it is also currently acknowledged that a strong persistence of probiotics in the GIT could generate dysbiosis via the excessive deconjugation of bile salts and/or degradation of intestinal mucus layer [24]. Anyhow, no serious adverse effects have been described in clinical trials, [25]. Accordingly, adhesion properties have been proposed as a crucial factor for the selection of new probiotic and they could easily be investigated using *in* *vitro* models of the intestine. These tools are also fundamental for the study of the interaction between probiotics, pathogens and human cells, such as the enhancing of the innate immune function and the inhibition of pathogen action [26].

Therefore, the objectives of the current paper were: (i) to identify new probiotic candidates in a collection of LAB isolated from table olives, through a comprehensive approach which would initially consider the safety features of the strains; and (ii) to establish their interaction with human epithelial cells. For this second purpose, the most suitable strains have been characterized with *in* *vitro* models of the gut, focusing attention on the inhibition of *L. monocytogenes* and stimulating the effect of intestinal barrier integrity.

## Materials and Methods

### 2.1 Bacteria and Sources of Isolations

Initially, 238 LAB were collected separately from brines and drupe surfaces of table olive fermentations carried out with the *Nocellara Etnea* variety. Most of this collection (191 strains) was isolated from two industrial processes conducted in small enterprises located in Sicily, as previously described [27]. These strains were identified as *Lb. plantarum* (182 isolates) and *Lb. pentosus* (9 isolates) species by means of multiplex PCR analysis of the *recA* gene, with species-specific primers for *L. pentosus*, *L. plantarum* and *L. paraplantarum*, according to the protocol described by Torriani et al. [28]. The remaining 47 isolates were recovered from experimental table olive fermentations carried out at both industrial and laboratory scale and followed as previously described [27], with the aim of determining the technological performances of potential starter cultures. This second collection was composed by: 24 isolates of *Lb. plantarum*, 7 *Lb. pentosus*, and 16 belonging to *Ln. mesenteroides*.

All the strains collected were purified by streaking and checked through Gram staining and catalase activity. Isolates were grown in Man Rogosa Sharp broth (MRS, Oxoid, Milan, Italy) for 24 h at 30°C and stored at - 80°C with 20% (w/v) of glycerol (Sigma, Milan, Italy).

### 2.2 Phenotypic Tests

Initial screening of the 238 isolated strains was performed according to the *in* *vitro* phenotypic tests described by Bautista-Gallego et al. [13]. Briefly, production of antimicrobial compounds, hemolytic activity, bile salt hydrolysis (BSH activity), autoaggregation, bacterial surface hydrophobicity and survival in a simulated human digestion process were used for the discrimination. In all tests the probiotic strains *Lb. rhamnosus* GG (LGG) and *Lb. casei Shirota* were used as reference controls.

The detection of bacteriocins production was assessed using the agar-well diffusion assay (AWDA) as described by Toba et al. [29] with some minor modifications. Briefly lawn of BHI (Oxoid) soft agar (10 g L^−1^) medium containing each indicator microorganism, namely *L. monocytogenes* FMCC B-128 and NCTC 10527, was poured onto Petri dishes. After solidification, 5 mm wells were made in the plates and filled with 50 μL of overnight BHI broth culture of each strain and left to diffuse for 30 min. After an overnight incubation at the optimum growth temperature for each indicator strains, the plates were examined for halos around the wells. For those strains, which showed the inhibition zones, the test was repeated adding a volume (4 μL) of proteinase K solution (25 mg mL^−1^; Sigma) in each well. The proteolytic enzyme was added in order to confirm the proteinaceous nature of the inhibitor compound.

To test for haemolytic activity, overnight lactobacilli broth cultures were streaked onto Columbia agar plates containing 50 g L^− 1^ of horse blood (Oxoid), and incubated for 48 h at 30°C. Then, the plates were examined for signs of α, β or γ-haemolysis.

The BSH activity was tested by using the plate assay described by Dashkevicz and Feighner [30]. Briefly, bile salt–MRS agar plates containing 5 g L^−1^ of bovine bile (Sigma) were inoculated with an overnight MRS culture, incubated at 37°C for 72 h, and then observed for colonies with precipitated bile salts.

Autoaggregation assays were performed according to the methodology described by Kos et al. [31]. The autoaggregation percentage was expressed as a function of time until it was constant, using the formula 1 − (A_t_/A_0_)×100, where A_t_ represents the absorbance measured at 600 nm (A600) at any time (1, 2, 3, 4 or 5 h), and A_0_ the absorbance at time t = 0 h. Final value of autoaggregation, after 5 h (AA), was used as index of the bacterial cells capability to aggregate among them.

Bacterial cell surface hydrophobicity was assessed by measuring microbial adhesion to hydrocarbons using the procedure described by Crow et al. [32] with some modifications. Briefly, bacteria growth at 37°C for 48 h were centrifuged (10 000×g for 5 min). The resulting pellet was washed twice in PBS, re-suspended in 3 mL of KNO_3_ 0.1 M and the A600 was measured (A_0_). One mL of o-xylene (Sigma) was then added to the cell suspension to form a two-phase system. After a 10 min pre-incubation at room temperature, the two-phase system was mixed by vortexing for 2 min. Then, the water and xylene phases were separated by incubation for 20 min at room temperature. The aqueous phase was carefully removed and the A600 was measured (A_1_). The percentage of the cell surface hydrophobicity (H) was calculated using the formula H = (1 − A_1_/A_0_)×100.

A simulated process of human digestion process was assessed performing consecutively a gastric and intestinal step of *in* *vitro* digestion. The gastric digestion step was simulated using the synthetic gastric juice described by Corcoran et al. [18]. The lactobacilli were grown for 24 h at 37°C, centrifuged and the pellet was washed with PBS. Initial count (T_0_) of the overnight culture was performed pleating serial dilution on MRS agar. Plates were incubated for 3–5 days at 37°C in microaerophilic conditions. Then, the bacteria were re-suspended in the synthetic gastric juice and incubated for 2 h at 37°C in an orbital shaker (∼ 200 rpm) to simulate peristaltic movements. Harvested cells from the gastric digestion step were washed in PBS and the enumeration of bacteria cells was performed as previously (T_g_). The pellet was then re-suspended in the same volume of the simulated intestinal juice, which was formulated using bile (3 g L^−1^, Oxoid) and pancreatin (0.1 g L^−1^, Sigma) in a buffer at pH 8.0 consisting of 50.81 g L^−1^ of sodium phosphate dibasic heptahydrate, 8.5 g L^−1^ of NaCl and 1.27 g L^−1^ of KH_2_PO_4_ [13]. After shaking at 200 rpm in an orbital shaker for 4 h at 37°C, the pellet was washed and then re-suspended in a volume of PBS and the enumeration on MRS plates after the intestinal step (T_i_) was performed as described above. Overall digestion survival (ODS) was obtained by comparison of the initial lactobacilli counts at the start of the simulated gastric digestion (T_0_) and those remaining at the end of the simulated intestinal digestion (T_i_). Data were expressed in percentage according to the formula: T_i/_T_0_× 100.

### 2.3 Chemometric Analysis and Selection

The final value of autoaggregation (AA), bacterial surface hydrophobicity (H) and ODS of the strains able to survive to the simulated digestion were used as input variables in hierarchical cluster analysis (HCA) based on Euclidian distances (single linkage distance). Considering these three variables, the probiotic strains LGG and *Lb. casei Shirota* were compared to the tested strains in the HCA. The HCA allowed the strains to be allocated into homogeneous groups according to their characteristics, identifying those deserving to be characterized subsequently by *in* *vitro* gut models [12].

Moreover, the physiological characteristics (ODS, AA, and H) of the selected strains, together with those of two reference probiotics, were subjected to ANOVA and Duncan’s test, in order to highlight any significant differences between the phenotypes. The data were analyzed using Statistica, ver. 7.0, (StatSoft Inc., Tulsa, USA).

### 2.4 Antibiotic Resistance

The micro-dilution broth test described by Argyri et al. [12] was used with slight modifications to test the antibiotic resistance of each strain selected in the previous phenotypic tests. The analysis was performed with eight antibiotics (ampicillin, gentamicin, kanamycin, streptomycin, erythromycin, clindamycin, tetracycline, chloramphenicol; Sigma), which were initially added at the species-specific breakpoint concentrations proposed by the European Food Safety Agency (EFSA) [33]. If resistant, the strains were progressively tested with higher antibiotic concentrations until their Minimum Inhibitory Concentrations (MICs) were found.

The strains were grown in MRS broth for 18 h at 37°C and, after a centrifuge step, the media were removed and the cells were washed twice with an isotonic solution (Ringer, Oxoid). Concentrations of bacteria suspensions were quantified through optical density (OD) measurement at 630 nm in order to standardize the inoculum at 7.0±0.5 log_10_ CFU mL^−1^. The suspensions were then inoculated in MRS broth (1% v/v), supplemented with each antibiotic, and incubated for 24 hours at 37°C. The experiment was performed in 96 microtiter well plates (Corning, New York, USA) and reference controls were performed by inoculating the bacteria in MRS not supplemented with antibiotics. Bacterial growth was monitored using an ELx880 microtiter plate reader (Savatec, Turin, Italy) by measuring OD at 630 nm after the incubation period. Three independent experiments were carried out and each assay was performed in duplicate.

The data were expressed as percentages of the bacterial growth in the antibiotic supplemented MRS (A_atb_) and compared with the growth in pure MRS (A_n_), using an A_atb/_A_n_× 100 formula. Strains with a percentage of growth ratio ≤5%were considered as not resistant.

### 2.5 *In vitro* Gut Models and Experimental Conditions

The experiments were performed using intestinal epithelial and monocyte/macrophage derived cell lines of human origin, named respectively H4 clone 1 (H4-1) and TLT. Both cell lines have been prepared and characterized at the Department of Microbiology, Biochemistry, Molecular Biology and Biotechnology at the Faculty of Agriculture and Life Sciences, University of Maribor (Maribor, Slovenia) [34]. H4 cell line was initially prepared from neonatal intestinal epithelia [35], [36] and further cloned to H4-1, which is characterized by developing high trans-epithelial electrical resistance. Macrophage cell line TLT was prepared from human blood-derived PBMC, isolated from a healthy donor [37]. Both cell lines were grown in an advanced Dulbecco Modified Eagle Medium (DMEM) (Gibco), supplemented with 5% foetal calf serum (Lonza, Basel, Switzerland), L-glutamine (2 mM, Sigma), penicillin (100 U mL^−1^, Sigma) and streptomycin (1 mgmL^−1^, Fluka, Buchs, Switzerland). The cell lines were routinely grown in 25 cm^2^ culture flasks (Corning, New York, USA) at 37°C in a humidified atmosphere containing 5% CO_2_ and 95% air, until confluent monolayers were obtained. The culture medium was changed routinely and once the cells reached confluence, after 3–4 days, they were subpassaged.

**Figure 1:**
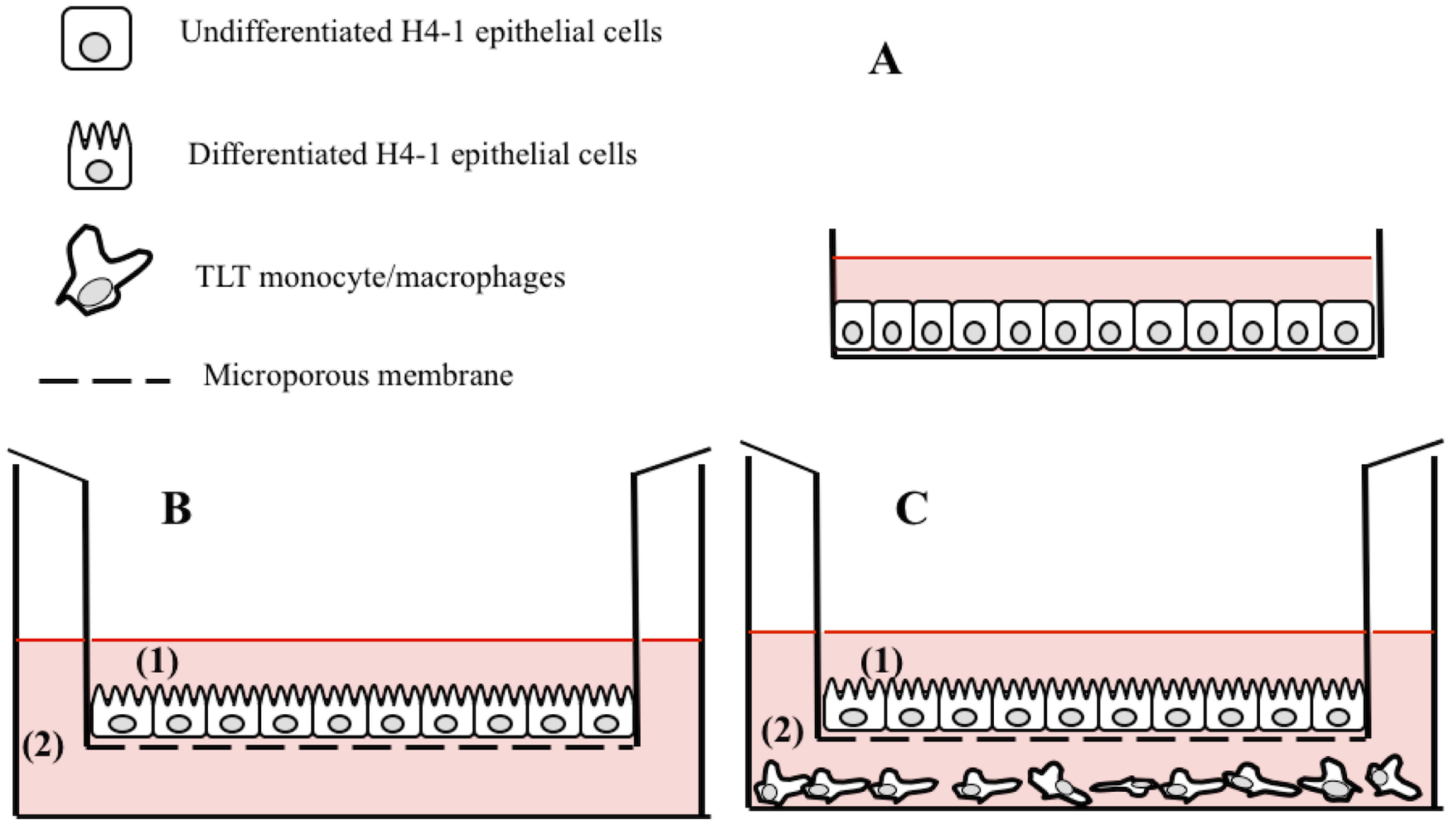
Experimental intestinal cell model settings used in this survey: (A) undifferentiated model with H4-1 epithelial cells grown on plastic surface; (B) functional 3D model where H4-1 were grown until the differentiation on microporous membrane; and (C) the complete 3D H4-1/TLT model with a differentiated layer of H4-1 in the apical compartment (1) and TLT monocyte/macrophages seeded in the basolateral side (2). In the models (B) and (C) the polarization of H4-1 cells was obtained after 14–15 days of incubation with regular changes of the media (cells initially seeded at a density of 50 000 cells per cm^2^), and confirmed by measuring the TEER. In the model (C) inserts with polarized H4-1 cells were transferred into wells that had been underlain with TLT cells (200 000 cells each well and growth for 24–48 h).

Different *in* *vitro* experimental set-ups of human gut epithelium were used: cell growing as an undifferentiated monolayer and in two functional 3D model, as shown schematically in Figure 1.

For the tests performed with the undifferentiated monolayer, 96-well flat bottom plates (Corning, New York, USA) were filled with a suspension of 100 000 cells/well (c/w) in DMEM supplemented with antibiotics and a serum, in order to obtain a complete and confluent monolayer after 24 hours of incubation at 37°C in a humidified atmosphere of 5% CO_2_ and 95% air (Fig. 1 A).

For the functional 3D model, the cells were seeded on polyester Transwell filter inserts with microporous (0.4 μm pore size, 12 mm, Corning) and placed in 12 well plates (22.1 mm, Corning) at a density of 50 000 cells/cm^2^. The filters were maintained with a volume of 0.5 mL in the apical compartment and 1.5 mL in the basolateral compartment. The cells were grown in the same medium and under the same conditions as those described above for 14–15 days with regular changes of the media, until functional polarization was reached. Polarization of the cells was established by measuring the trans-epithelial electrical resistance (TEER) with the Millicell Electrical Resistance System (Millipore, Bedford, MA), as described previously [37]. At the same time, TLT cells were seeded in 12 well plates (200 000 c/w) and further incubated until confluence (24–48 hours) (Fig. 1B). In order to establish the complete 3D H4-1/TLT model, inserts with H4-1 cells were transferred into wells that had been underlain with TLT cells (Fig. 1C).

All the selected strains tested with the cell models were cultured for routine use in MRS broth and grown for 18 h at 37°C, in order to test them in an exponential phase. In all the experiments performed with human cells, the initial concentration of bacterial strains was determined by OD at 595 nm and all the suspensions were set to the same initial count using an internal standard curve. Before the experiment, the bacteria was washed twice in PBS and then resuspended in DMEM w/o antibiotics and serum. Moreover, in the human gut experimental set-ups, the H4-1 epithelial cells were washed three times with PBS before the addition of the bacteria, in order to eliminate any traces of the antibiotics.

The results of the experiments with human cells were expressed as the mean and standard error mean (SEM) of at least three experiments with duplicate or triplicate determinations. Statistical analyses were performed with Statistica, ver. 7.0, (StatSoft Inc., Tulsa, USA). In order to assess the overall variation and differences between the multiple groups, the numerical values were analyzed by ANOVA (One way-Analysis of Variance) and with Duncan’s post-hoc test. The Student’s *t*-test was also used to compare individual groups with the control or with other individual groups. In all experiments performed with *in* *vitro* gut models LGG strain was used as the probiotic reference strain (positive control).

### 2.6 Cellular Metabolic Activity

The cellular metabolic activity with 3-(4,5-dimethylthiazole-2-yl)-2,5-phenyl tetrazolium bromide (MTT; Sigma) was assessed in order to evaluate the potential cytotoxic effect of the selected strains towards H4-1 and TLT human cells. Undifferentiated monolayer and the complete 3D model (H4-1/TLT) were incubated with heat inactivated and live bacteria, respectively. In short, overnight bacterial cultures were set to 8.0±0.5 log_10_ CFU mL^−1^, washed twice with PBS and heat inactivated at 95°C for 30 minutes [38]. This inactivation was made to avoid a misinterpretation of the results, since the MTT reduction may be lead by bacterial enzymes as well [39]. After the inactivation, the bacteria were resuspended in fresh DMEM and added to the H4-1 cells growing in the monolayer. The heat inactivated bacteria and H4-1 cells were co-incubated for 24 h at 37°C in a humidified atmosphere containing 5% CO_2_ and 95% air. In order to evaluate cytotoxicity towards the TLT cells, an 8.0±0.5 log_10_ CFU mL^−1^ suspension of live bacteria, prepared in DMEM w/o antibiotics and serum, was seeded in the upper compartment of the 3D model (H4-1/TLT), but not in direct contact with the macrophages, in order to simulate the real conditions of the human gut. The live bacteria were incubated in the 3D model (H4-1/TLT) for 24 h at 37°C in a humidified atmosphere containing 5% CO_2_ and 95% air.

After 24 h of incubation, the supernatant was removed and H4-1 and TLT cells were put in contact with DMEM w/o phenol red supplemented with 5 mg mL^−1^ of MTT (Sigma) for 3 h. At the end of MTT degradation, the supernatant was gently removed and the formazan on the bottom was dried and solubilized with a 0.04% HCl solution in isopropanol (Sigma).The cellular metabolic activity of the H4-1 and TLT cells was detected spectrophotometrically at 570 nm [40] and data were expressed by means of the following equation: 100- (OD of test sample/OD of blank×100). Cells not treated with bacteria were used as blanks.

### 2.7 Adhesion Assay

The adhesion ability of the selected strains was assessed on both the undifferentiated monolayer and on the functional 3D model of the H4-1 cells. The bacterial strains were seeded in an 8.0±0.5 log_10_ CFU mL^−1^ concentration and incubated for 90 min in a modified atmosphere of 5% CO_2_ and 95% air. The inoculum of each tested strain was quantified in each experiment using the standard CFU method. The H4-1 cells were washed five times with PBS and the cells were homogenized with a Triton-X solution (0.25% in PBS; Sigma). After 30 minutes of incubation, the solution with released bacteria was serially diluted and plated on MRS agar. The plates were incubated for 48 hours at 37°C in microaerophilic conditions. Adhesion ability was expressed as the percentage ratio between the counts initially seeded and the counts after the washing steps (CFU mL^−1^). In parallel, assays were carried out on each bacterial strain to exclude any potential harmful effect on the survival of bacteria due to the treatment with the 0.25% Triton-X solution.

### 2.8 *In vitro* Adhesion and Invasion of *Listeria monocytogenes*

The capability of the LAB strains to inhibit *in* *vitro* the adhesion and invasion of *L. monocytogenes* WT (collected at the Faculty of Agriculture and Life Sciences, University of Maribor) was established on the H4-1 cells according to the method described by Corr et al. [4], with some minor modifications. Trials were performed with an undifferentiated monolayer treated with probiotic strains. The treatment was carried out by seeding 8.0±0.5 log_10_ CFU mL^−1^ of each selected LAB onto the cell model. The human cells and LAB were then incubated for 90 min in a modified atmosphere containing 5% CO_2_ and 95% air and washed five times with PBS, as described in the adhesion assay, in order to remove non adherent bacteria. *L. monocytogenes* was added in the next step. A multiplicity of infection (MOI) 10 was used for the ratio between the pathogen and the human cells, as suggested by Yamada et al. [41] for non tumorigenic cell lines of animal origin. This MOI was also established as being optimal for the H4-1 cells through a cytoxicity assay. In both assays, an 18 h pre-cultivated pathogen was inoculated in Nutrient Broth (Oxoid), incubated at 37°C for 13 h, washed twice with PBS, resuspended in DMEM w/o antibiotics, diluted in order to reach the appropriate bacterial count (7.0±0.5 log_10_ CFU mL^−1^) and then inoculated on the cell monolayer surface. After 2 h of co-incubation at 37°C, the monolayers were washed three times with PBS in order to remove any unattached pathogenic bacteria. The cells with attached *L. monocytogenes* were lysed in PBS containing 0.25% Triton-X, as described above for the adhesion assay. Counts were performed by means of the CFU method, plating the dilutions on Listeria Selective Agar (Fluka, St. Gallen, Switzerland) and incubating them at 30°C for 48 h. The result of the adhesion inhibition assay can be considered representative of the overall action of the strains, both against the *L. monocytogenes* attached to the cell membranes and against the pathogen present inside the cell cytosol.

As far as the invasion assay is concerned, after the final washing step to remove non-adhered *L. monocytogenes* cells, the DMEM media supplemented with 50 μg mL^−1^ gentamicin sulfate (Sigma) was added to the cell monolayers and further incubated for 2 h at 37°C to kill all the extracellular bacteria [4]. The monolayer was washed three times, the cells were lysed with Triton-X 0.25% and the bacterial counts were performed as described above. The action of the strains against pathogen invasion of the human cells was evaluated specifically in this second experiment.

The results of the adhesion and the invasion assays were expressed as percentage ratios of the *L. monocytogenes* recovered from the treated wells and the count from the wells not treated with LAB strains (CFU mL^−1^).

### 2.9 Trans-epithelial Electrical Resistance (TEER) as a Measurement of the Epithelial Barrier Integrity

The bacterial effect on the epithelial barrier was evaluated by measuring the TEER using the Millicell Electrical Resistance System, as described by Jensen et al. [42], with some minor modifications. A functional 3D model of the H4-1 cells was constructed as previously reported. An optimal functional polarity was developed after 14 days of growth on the membrane, and an average TEER of 740±100 Ω/cm^2^ was reached. On the day before the experiment, the filters were washed with PBS to remove traces of the antibiotics and the cell media was changed to the original media without antibiotics. TEER was measured before the addition of bacteria (TEER_t0_), and after 1, 3, 5, 7, 18 and 24 h of incubation (TEER_tn_). The bacterial effect on TEER was tested with 10^7^ CFU mL^−1^ of inoculated bacteria. *L. monocytogenes* WT and DMEM were used in the same concentration as the negative and positive controls, respectively.

The TEER (TEER_tx_/TEER_t0_×100) ratio was calculated and the bacterial effect on the epithelial barrier over time was compared for the investigated strains.

## Results

### 3.1 Selection of the most Promising Strains

In the present study, 238 LAB strains collected from different *Nocellara Etnea* green table olive fermentation processes were screened *in* *vitro* for their phenotypic features related to probiotic traits. The results of the phenotypic screening of all the isolates are summarized in Table S1.

With regard to the autoaggregation indices, the strains exhibited a normal distribution, with the largest number of the observations located in the interval between 10 and 20%. Furthermore, *Lb. plantarum* strains showed a higher autoaggregation phenotype (23% on average) with respect to the other two species isolated, *Lb. pentosus* and *Ln. mesenteroides* (19% in average). *Lb. plantarum* O1T90E, S1T30B and O2T60D showed the highest autoaggregation values (more than 50%). However, most of the isolates showed hydrophobicity values of less than 40%, with a strongly skewed distribution of the observations tending towards the lower values. However, two strains of *Lb. plantarum* showed values of over 90% (S3T60C and S2T30B).

As far as the potential inhibition of the selected indicator pathogensis concerned, the first AWDA highlighted 22 strains of *Lb. plantarum* and the *Ln. mesenteroides* FO50O that were able to inhibit the growth of *L. monocytogenes* FMCC B-128 and NCTC 10527. However, the confirmation screening, with proteinase K, attributed this antagonist effect to the production of organic acids (Table S1). It therefore resulted that no strains were able to produce bacteriocins and none showed BSH or hemolytic activities (data not shown).

**Figure 2:**
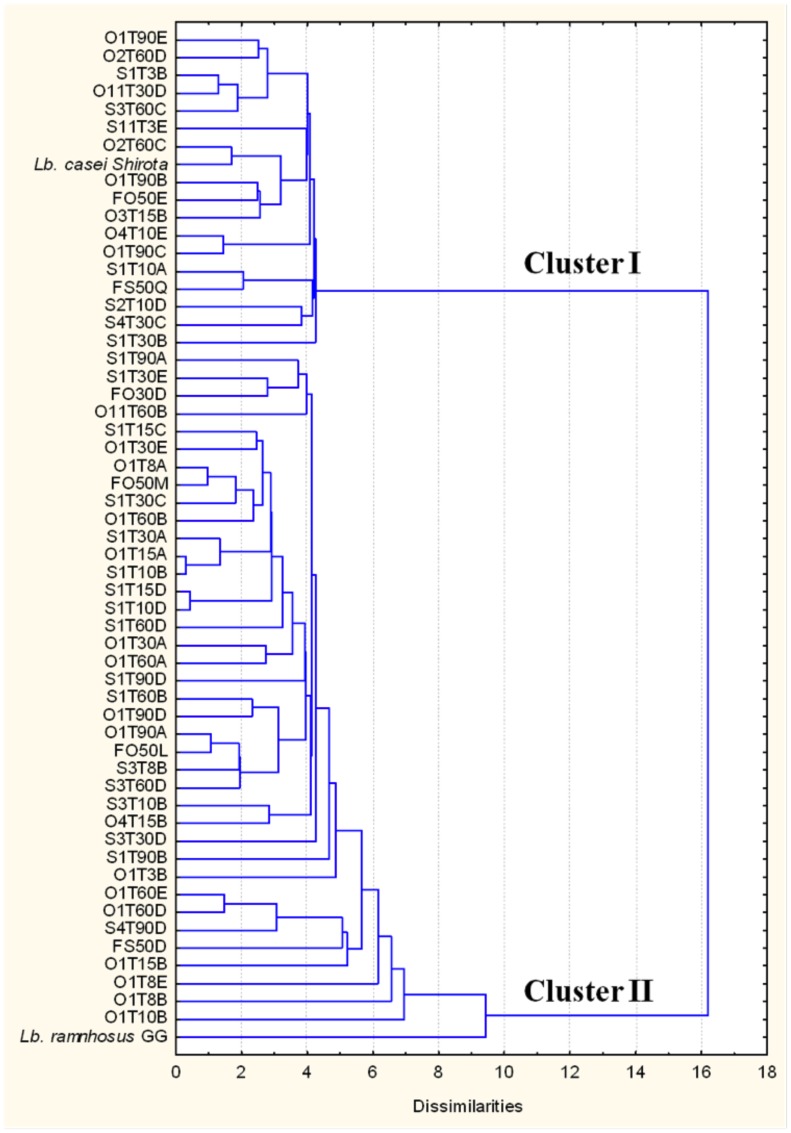
Dendrogram resulting from the hierarchical cluster analysis (HCA) of the 55 strains resistant to the simulation of digestion. The calculation of the dissimilarity between the cases is based on three independent indices expressed in percentage: ODS, autoaggregation (AA) and hydrophobicity (H). *Lb casei Shirota* and *Lb. rhamnosus* GG (LGG) were inserted in the HCA as reference probiotic controls.

Regarding the effect of the digestion simulation over the strains (gastric and pancreatic transits), we considered as not resistant those which presented after the two passages undetectable viable counts (<10 CFU mL^−1^), corresponding to an ODS value of 0.00001%. Overall, 55 strains (23.1% of the tested bacteria) were resistant to the simulated digestion process (Table S1), Using the ODS rates, together with the percentages of hydrophobicity and autoaggregation, hierarchical cluster analysis highlighted two well-defined groups (Fig. 2). The lower group (cluster II) was larger and contained strains with less resistance to simulated human digestion together with a weak auto-aggregating and hydrophobic phenotypes. The reference strain, *Lb. rhamnosus* GG, which showed a poor probiotic potential in the tests, was also allocated to this cluster, whereas *Lb. casei Shirota* was grouped in cluster I.

**Figure 3:**
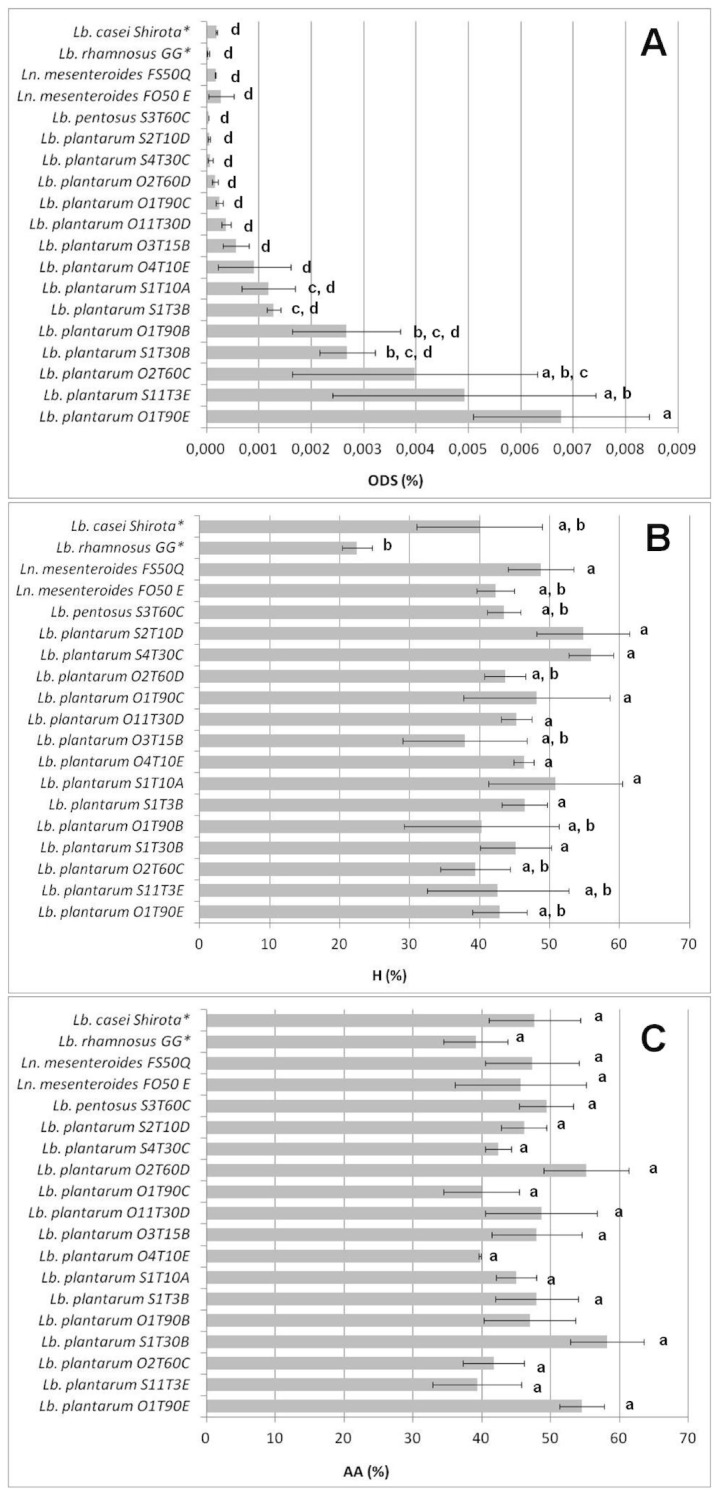
Overall digestion survival (ODS %) index (A), hydrophobicity (H %) index (B), and autoaggregation (AA %) index (C) of the 17 selected strains. The data are the means of three independent experiments ± SEM and the bars marked with different letters (a,b,c,d) indicate a significant difference at *P*<0.05 (ANOVA with Duncan’s test as post hoc). (*): reference strains for the experiment.

This group collected 17 strains with the most promising phenotypes, and these were therefore selected and further characterized for antibiotic susceptibility and tested with human cell models. The phenotypic features (ODS, H, AA) of these strains are shown in Figure 3. The majority of them belonged to the *Lb. plantarum* species (15 isolates), moreover, two strains of *Ln. mesenteroides* coded FS50Q and FO50E, and the *Lb. pentosus* S3T60C were included. Focusing attention on the ODS (Fig. 3 A), it was observed that three strains, O1T90E, S11T3E and O2T60C, showed values of over 0.003%, which were significantly higher than the positive reference strains *Lb. casei Shirota* and LGG (*P*<0.05), and the highest survival ratio was shown for the *Lb. plantarum* O1T90E, with an overall survival rate of 0.00677%. As far as the hydrophobicity (Fig. 3B) and autoaggregation (Fig. 3C), no significant differences were observed among the 17 tested candidates (*P*>0.05).

### 3.2 Antibiotic Resistance

The resistance levels detected for the selected strains are shown in Table 1. It can be observed that ampicillin, erythromycin, clindamycin and chloramphenicol completely inhibited the growth of all the strains at the breakpoint concentrations proposed by the EFSA. The same behavior was shown by streptomycin for the *Lb. pentosus* and *Ln. mesenteroides* species at the suggested MICs, whereas the EFSA does not require any specific limit for these antibiotics in the case of *Lb. plantarum* strains. Tetracycline inhibited all the strains, with the exception of *Lb. pentosus* S3T60C, which was only completely inhibited at 24 μg mL^−1^, that is, three times the breakpoint proposed for facultative heterofermentative lactobacilli such as *Lb. pentosus*. The S3T60C strain was even resistant to the suggested kanamycin MIC, which, together with gentamicin, could not inhibit the majority of the *Lb. plantarum* isolates. In 15 strains of *Lb. plantarum*, only S2T10D was completely inhibited at 64 μg mL^−1^ of kanamycin, whereas 5 strains were able to grow to concentrations of 192 μg mL^−1^ and the remaining 8 strains showed an MIC of 296 μg mL^−1^. Moreover, the O1T90E, S1T3B, O4T10E and S11T3E strains, resulted to be resistant to both gentamicin and kanamycin. Finally, the *Ln. mesenteroides* strains were susceptible to all the antibiotics tested at the MICs reported as breakpoints.

**Table 1:** Minimum Inhibitory Concentrations (MICs) detected for the selected strains towards the antibiotics: ampicillin (A), gentamicin (G), kanamycin (K), tetracycline (T), streptomycin (S), erythromycin (E), clindamycin(C), chloramphenicol (Ch).

Strains	MICs (μg mL^−1^)							
	A	G	K	T	S	E	C	Ch
*Lb. plantarum*:								
O1T90C	<2	<16	296*^R^*	<32	n.r.	<1	<1	<8
O11T30D	<2	<16	296*^R^*	<32	n.r.	<1	<1	<8
O1T90E	<2	32*^R^*	296*^R^*	<32	n.r.	<1	<1	<8
S1T3B	<2	32*^R^*	296*^R^*	<32	n.r.	<1	<1	<8
O1T90B	<2	<16	296*^R^*	<32	n.r.	<1	<1	<8
O2T60C	<2	<16	192*^R^*	<32	n.r.	<1	<1	<8
S1T10A	<2	<16	296*^R^*	<32	n.r.	<1	<1	<8
O4T10E	<2	32*^R^*	192*^R^*	<32	n.r.	<1	<1	<8
S4T30C	<2	<16	192*^R^*	<32	n.r.	<1	<1	<8
S1T30B	<2	<16	192*^R^*	<32	n.r.	<1	<1	<8
O3T15B	<2	<16	296*^R^*	<32	n.r.	<1	<1	<8
O2T60D	<2	<16	192*^R^*	<32	n.r.	<1	<1	<8
S11T3E	<2	32*^R^*	296*^R^*	<32	n.r.	<1	<1	<8
S2T10D	<2	<16	<64	<32	n.r.	<1	<1	<8
*Lb. pentosus*:								
S3T60C	<4	<16	192*^R^*	24*^R^*	<64	<1	<1	<4
*Ln. mesenteroides*:								
FS 50 Q	<2	<16	<16	<8	<64	<1	<1	<4
FO 50 E	<2	<16	<16	<8	<64	<1	<1	<4

(*^R^*): resistance according to the EFSA breakpoints [33].

(n.r.): test not required [33].

### 3.3 Modulation of Cellular Metabolic Activity

**Figure 4:**
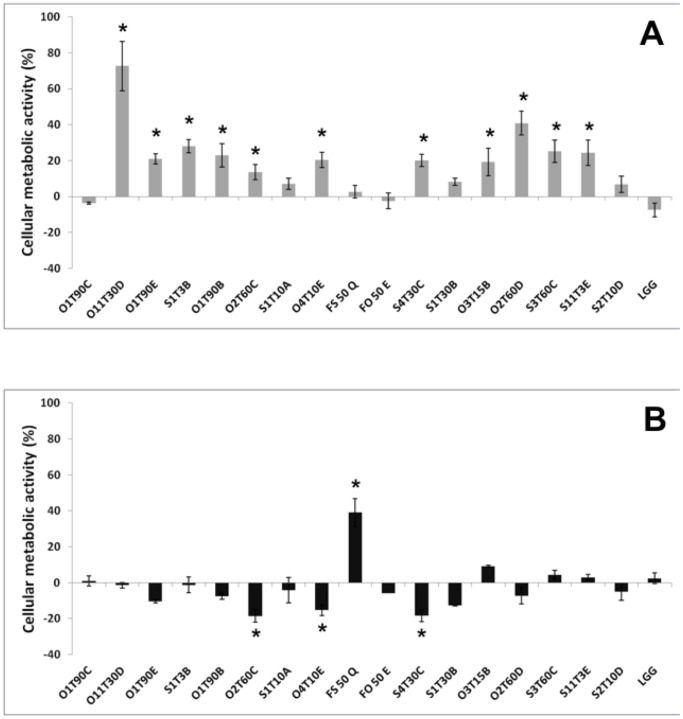
Variation of the cellular metabolic activity in H4-1 (A) and TLT (B) cells, respectively incubated with heat inactivated and live bacteria for 24 h. Undifferentiated model was used for the H4-1, whereas a complete 3D model (H4-1/TLT) was developed to assess the cellular metabolic activity in TLT. The data are expressed as mean ratios (% -100; ±SEM) of absorbance in the treated well (OD of test sample) and those not treated (OD of blank) with bacteria: 100- (OD of test sample/OD of blank×100). The asterisks (*) highlight significantly different values (Duncan’s test; *P*<0.05) compared to the reference strain *Lb. rhamnosus* GG (LGG).

All the inactivated bacteria strains left the cellular metabolic activity of the H4-1 cells unchanged or even improved (Fig. 4A). The reference probiotic, LGG, did not enhance the metabolic activity of H4-1cells, and similar behavior was observed for *Lb. plantarum* O1T90C and S1T10A and for *Ln. mesenteroides* FS50Q and FO50E. All the other tested LAB determined an increasing in the metabolic activity of the H4-1 cells from 15 to 70%, thus showing significantly higher results than the control LGG (*P*<0.05).

Concerning the experiment performed incubating live bacteria with TLT cells in the 3D model (H4-1/TLT), it can be observed in Figure 4B that *Ln. mesenteroides* FS50Q caused a remarkable increase in the metabolic activity, with significantly higher values (*P*<0.05) than for the probiotic reference (LGG). On the other hand, *Lb. plantarum* strains O2T60C, O4T10E, and S4T30C significantly decreased the TLT metabolic index with respect to LGG (*P*<0.05).

### 3.4 Adhesion Capability

**Figure 5:**
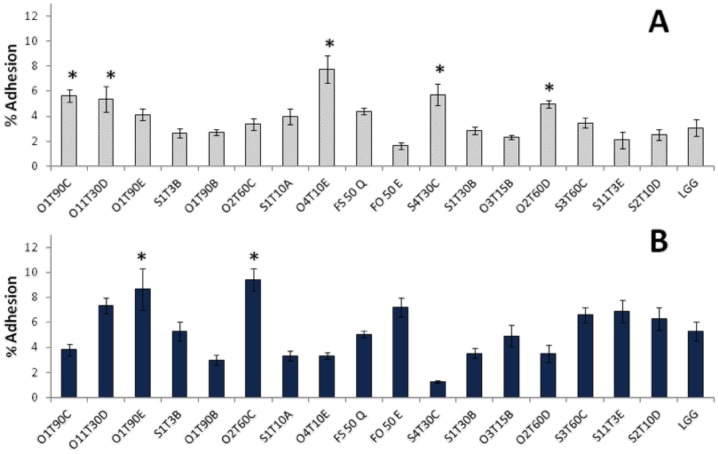
Adhesion profile of the LAB to the undifferentiated monolayer (A) and 3D functional model of H4-1 (B), expressed as the mean ratio (%) between the bacteria recovered from the human cells after incubation and the washing steps and the initial bacterial count (8.0±0.5log_10_ CFU mL^−1^) of the inoculum (n = 3; ± SEM). The marked bars (*) indicate significantly (*P*<0.05) higher values (ANOVA with Duncan’s test as post hoc) to the reference strain *Lb. rhamnosus* GG (LGG).

The adhesion capacity of the 17 most promising strains was variable and generally low (from 1% to 10%). Moreover, an enhancement of adhesion during the step from the undifferentiated to the 3D model of the H4-1 cells was observed for most of the strains (14) (Fig. 5). The overall average adhesion to the undifferentiated cells was 3.82%, whereas adhesion to the apical side of the cells reached 5.27% in the 3D functional model. This behavior was statistically significant for the *Lb. plantarum* O2T60C and *Ln. mesenteroides* F050E strains (*P*<0.05). However, *Lb. plantarum* S4T30C adhered significantly less in the 3D model (*P*<0.05).

Focusing attention on the experiment performed with the undifferentiated model, it was observed that 5 strains (O1T90C, O11T30D, O4T10E, S4T30C and O2T60D) showed a higher adhesion ratio than the reference probiotic LGG (*P*<0.05). These strains did not confirm their adhesive potential in the 3D model, in which the O1T90E and O2T60C strains showed the best adhesiveness, with 8.67% and 9.41%, and were significantly higher than LGG (*P*<0.05).

### 3.5 Strains Effect Towards Adhesion and Invasion of *L. monocytogenes*

**Figure 6:**
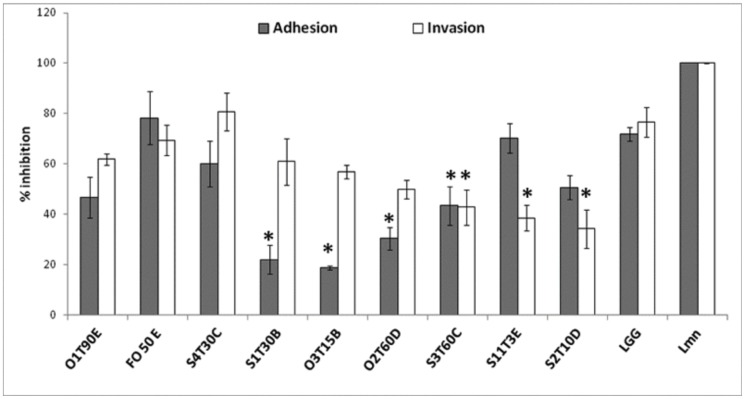
LAB strains that are able to inhibit the adhesion and invasion of *L. monocytogenes* (MOI:10). The data are the average (± SEM) of three independent experiments and are expressed as percentage ratios of the recovered pathogens in the treated wells with respect to those recovered from the non -treated well (Lmn). The marked bars (*) indicate significantly lower counts (log_10_ CFU mL^−1^) than those of the cells infected with *L. monocytogenes* alone (Student’s *t*-test, *P*<0.05).

In order to identify which strains could inhibit epithelial cell infection with *L. monocytogenes in* *vitro,* adhesion and invasion assays were carried out in parallel. Ten strains, including the reference probiotic LGG, showed potential inhibition (Fig. 6). The *Lb. plantarum* strains S1T30B, O3T15B, O2T60D, together with *Lb. pentosus*S3T60C, significantly reduced the adherence of the bacteria to the monolayer surface with respect to the untreated controls (*P*<0.05). *Lb. pentosus* S3T60C as well as the *Lb. plantarum* strains S11T3E and S2T10D were even able to significantly (*P*<0.05) reduce the intracellular invasion of the pathogen, by 57%, 61% and 66%, respectively.

### 3.6 Epithelial Barrier Integrity

**Figure 7:**
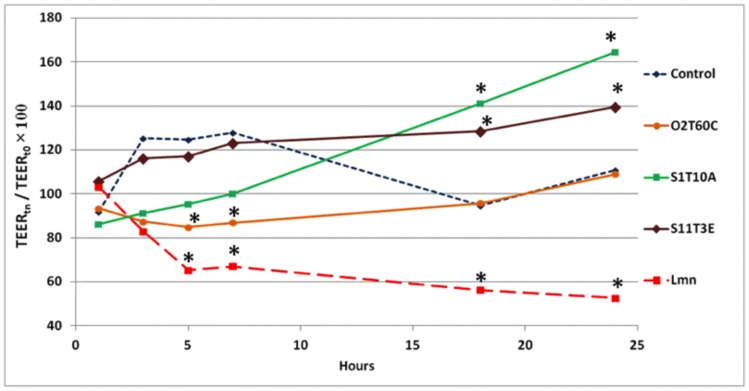
Time dependent TEER of the polarized H4 monolayer exposed to the strains: O2T60C, S1T10A, S11T3E and *L. monocytogenes* WT (Lmn). The untreated monolayer was used as a reference control. The bacterial effect on TEER dynamics was tested with 10^7^ CFU mL^−1^ of bacterial inoculum. The data of three independent experiments are expressed as the ratio (%) of TEER at time n in relation to the initial value (t_0_). The marked bars (*) indicate significantly different values (*P*<0.05) from the untreated control (ANOVA with Duncan’s test as post hoc). The complete dataset of the experiment (mean ± SEM), with the results of the statistical analysis, is reported in Table S2.

The measure of TEER in polarized *in* *vitro* gut models is commonly used to verify the cell monolayer integrity after the incubation with probiotic microorganisms [42], [43]. Overall, after 3 and 5 hours of incubation, the time dependent TEER values of cells co-inoculated with bacteria were lower than the control, but not significantly. The O2T60C strain instead showed a significant reduction in the TEER from the 5^th^ to the 7^th^ hour of incubation, compared to the untreated control (Fig. 7). As expected, the negative control, *L. monocytogenes* WT, quickly degenerated the junctions between the cells, and this resulted in a significant reduction in the TEER values from the 5^th^ hour of incubation until the end of the trial. Only the *Lb. plantarum* strains S1T10A and S11T3E showed a significant increase in the TEER values, compared to the control, after 18and 24 hours of incubation (*P*<0.05). The data of all the tested strains are shown in Table S2.

## Discussion

In this paper, a collection of 238 LAB strains, isolated from different green table olive fermentation processes, has been screened progressively, using as selective phenotypic traits the autoaggregation, the adhesion to hydrocarbons (hydrophobicity), and the capability to survive in the simulated GIT conditions [13], [22], [44]–[46].

Other interesting phenotypic traits, such the production of bacteriocins and BSH activity were also assessed, but no strain showed these properties. Even though BSH activity is correlated to a decrease in cholesterol, it is not completely clear whether BSH activity is a desirable property for probiotics, since large amounts of de-conjugated bile salts may have undesirable effects on the human host [12], [13]. As far as direct pathogen inhibition is concerned, many probiotic LAB have been observed to produce anti-microbial substances, and mainly organic acids, especially lactic and acetic acids [47]. Recently, the effective production of bacteriocins in the human intestinal conditions has been confirmed by *in* *vitro* and *in* *vivo* trials [48]. However, bacteriocins actively produced *in* *vitro* may not necessarily be sufficiently high in quantities, or at all, within the GIT [49]. Therefore, the production of bacteriocins might not be considered a fundamental tract for a new probiotic candidates.

The ability to autoaggregate and the cell surface hydrophobicity, are instead often suggested as suitable ways of identifying potential adherent bacteria, because related to the adhesiveness upon the intestinal epithelial layer [50], [51]. As far as the ODS is concerned, the *in* *vitro* resistance to the simulated GIT conditions is considered one of the most important feature for a probiotic candidate, since a microorganism without this prerequisite cannot develop its beneficial effect inside the gut [22]. For this reason, these two indices were used as variables, together with the ODS, in the HCA performed on the 55 strains able to survive to the simulated digestion. The dendrogram resulting from the HCA (Fig. 2) showed an opposite collocation of the two reference strains included in the analysis, *Lb. casei Shirota* and LGG, mainly due to the low ODS and AA rates of the LGG. A similar result was achieved in the study by Argyri et al. [12], in which the HCA was performed without autoaggregation and hydrophobicity variables and considering only strain resistance to simulated digestion. Anyhow, the chemometric analysis performed allowed the number of isolates to be reduced from the initial 55 strains to a final set of 17 probiotic candidates, which, considering together these three variables, highlighted the best phenotypic profiles.

As far as the phenotypic features of this set of probiotic candidates is concerned (Fig. 3), a limited difference in the autoaggregation and hydrophobicity values was observed among the strains, whereas a broad range of ODS values was detected with all values lower than 0.01%. However, as already reported by Bautista-Gallego et al. [13], the simulation of human digestion performed in the present study significantly reduced the viability of LAB, and a survival ratio of 0.008% could be considered as an appreciable result.

The 17 selected probiotic candidates were therefore tested for evaluating potential resistance to antibiotics or cytotoxic effect towards human cells. Concerning the antibiotic resistance, total inhibition for erythromycin, a Gram-positive spectrum antibiotic, which is known for its effective inhibition of LAB [52], was observed for the species-specific MICs suggested by the EFSA (Tab. 1). All the selected LAB were susceptible to β-Lactam antibiotic ampicillin and to the broad spectrum antibiotics clindamycin, chloramphenicol and tetracycline, with the exception of the latter against the *Lb. pentosus* strain S3T60C. Numerous strains were instead resistant to kanamycin and gentamicin, two aminoglycoside antibiotics. This resistance had already been observed for *Lb. plantarum* strains isolated from fermented vegetables [53] and as suggested by Hummel et al. [54], it could be considered as an intrinsic feature of LAB, that could be attributable to the absence of cytochrome-mediated electron transport, which mediates drug uptake. Therefore, membrane impermeability plays an important role in this intrinsic aminoglycoside resistance and it has been proved that the sensitivity of LAB is increased in the presence of bile, as in the intestine [55]. In light of these reports, the low susceptibility to kanamycin and gentamicin can be considered acceptable. Always in the context of strain safety, cytotoxicity has been evaluated towards human cells through an MTT assay. This is a well-known tool for the evaluation of cellular metabolic activity [56], since this reaction occurred in both the mitochondrial respiratory chain and outside the mitochondria, [57]. The heat-killed bacteria generally enhanced the viability of the H4-1 cells (Fig. 4A). A slow decreasing effect in the cellular metabolic activity of the TLT macrophages was shown by three *Lb. plantarum* strains (Fig. 4B).

After the safety assessments, the 17 selected LAB were further tested in adhesion to intestinal cell line experiments, in order to indirectly measure their ability to colonize the intestinal tract. In this study, it was decided to only focus attention on the interaction between the selected strains and the H4-1 cells, without considering colon tumorigenic cells, for example, Caco-2 and HT-29, since they have a tumorigenic phenotype that distinguishes them from normal gut epithelia [34], [37]. As far as the results of our selected LAB are concerned, a better capability to adhere to the polarized monolayer (3D model) than to the normal monolayer was noted (Fig. 5). Both in the undifferentiated and 3D model, highly variable adhesion values were observed (from 1 to 10%), which confirmed that the adhesion was a strain-specific characteristic [51]. The adhesion values recorded for the control strain LGG are in agreement with the results obtained by others [58] on H4-1 undifferentiated cells. In a similar study, *L. plantarum* isolated from table olives showed the same adhesion range when tested on Caco-2 [11]. However, it should pointed out that the adhesiveness of a selected probiotic strain or in probiotic candidates may not guarantee its probiotic health promoting activity in the host [59]. Moreover it is more and more accepted that probiotics can provide beneficial effects even without true colonization of the GIT [60].

The modulation of gut microbiota, which causes a reduction in harmful microorganisms, is one of the main features of bacterial strains that can be defined as probiotic [61]. The characterization of the 17 probiotic candidates was therefore followed by testing their capability to inhibit both adhesion and invasion of *L. monocytogenes* in undifferentiated human gut model. This facultative intracellular bacterial pathogen has evolved a number of mechanisms to exploit host processes, growing and spreading from cell to cell without damaging the host cell [62]. As shown (Fig. 6), 9 strains, that could concurrently inhibit the adhesion and the invasion of *L. monocytogenes* in the H4-1 undifferentiated cell model, were identified. These two potential capabilities did not seem related in the remaining 8 potential probiotics. For example, the O1T90C strain determined a significant reduction in *L. monocytogenes* adhesion compared to the untreated monolayer (*P*<0.05), but, at the same time, it caused an increase in the pathogen invasion to the H4-1 monolayer (data not shown). Moreover, the inhibition of *L. monocytogenes* adhesion did not depend on the capability of the LAB to adhere to human cells. As support of this finding, it was observed that strains S1T30B, O3T15B and S3T60C significantly reduced pathogen adhesion on the undifferentiated monolayer compared to the untreated control (*P*<0.05). However, these strains showed poor attachment to the H4-1 cells (Fig. 4). The non-correlation between the attachment capability of LAB and their activity against *L. monocytogenes* adhesion has already been highlighted by Koo et al. [63] and Botes et al. [64] using Caco-2 cells as a model. Accordingly, *L. monocytogenes* adhesion inhibition cannot be attributed solely to the simple competition between pathogens and LAB for the attachment sites on the cells.

As for the invasive action of *L. monocytogenes*, only three strains were able to reduce the pathogen invasion in a significant manner (*P*<0.05), compared to the control (Fig. 6), and of these, only *Lb. pentosus* S3T60C was able to reduce adhesion of the pathogen as well. Compared to the results obtained in other similar studies [4], [9], [63], the invasion inhibition shown by the S3T60C, S11T3E and S2T10D strains can be considered interesting, since they reduced the invasion of *L. monocytogenes* in the undifferentiated cells by over 60%. Since the pathogenicity of *L. monocytogenes* is mainly determined by its capability to cross the cell plasma membrane and remain alive and active inside the cytosol [65], results obtained by using S3T60C, S11T3E and S2T10D strains can be considered interesting and worthy of further in-depth studies. *L. monocytogenes* invasion inhibition mechanisms might be caused by the modulation of human cells or by direct inhibition of the pathogen, by acidification or the production of antimicrobial compounds. The second suggestion can be excluded for S3T60C and S2T10D strains, since, in previous AWDA, they did not inhibit *L. monocytogenes* growth, either through bacteriocin production or through acidification, whereas *Lb. plantarum* S11T3E was able to inhibit the growth of the pathogen through the production of organic acids (Table S1).

As far as the cell modulation activity of the strains is concerned, the potential enhancement of epithelial integrity was investigated by measuring the TEER. Improvements in barrier integrity are associated with changes in tight junctions (TJ) via changes in TJ protein expression and distribution. Strains of *Lb. plantarum* have been shown to regulate human epithelial TJ proteins *in* *vivo* [66] and *in* *vitro* [67]. In the present study, two strains of *Lb. plantarum* (S11T3E and S1T10A) enhanced the values of TEER compared to the control (*P*<0.05) when applied in a differentiated 3D model (Fig. 7). As already demonstrated in another study [43], the pathogen *L. monocytogenes* significantly reduced the integrity of the epithelial layer from almost the very beginning of the incubation period. Like other pathogens, *L. monocytogenes* may alter TJ permeability to allow its own translocation through the epithelial barrier [68]. In light of this, *Lb. plantarum* S11T3E might be prevented from invading *L. monocytogenes* through an amelioration of the integrity of the epithelial barrier. However, *L. monocytogenes* is able to directly cross the cell membrane and hence the reinforcement of the paracellular spaces may not be the only reason for the inhibitory action of S11T3E, which then needs further studied.

## Conclusions

In the current study, an extensive collection of autochthonous LAB from table olive fermentations has been screened, focusing on their tolerance to the hostile environment of the stomach and intestine, together with other suitable phenotypic features, such as high autoaggregation and hydrophobicity. A cluster analysis on their phenotypes has allowed the LAB set to be reduced to 17 strains belonging to the *Lb. plantarum*, *Lb. pentosus* and *Ln. mesenteroides* species. None of these selected strains showed resistance to broad spectrum antibiotics and they were confirmed to be safe and not cytotoxic when in contact with H4-1 human epithelial cells. Therefore, all the strains were characterized in relation to their probiotic potential using *in* *vitro* undifferentiated and 3D functional models of the H4-1 human intestinal epithelial cell line.

In conclusion, the S11T3E strain belonging to the *Lb. plantarum* species has overall shown the best probiotic performance, due to its high resistance to simulated gastric digestion, an increased transepithelial resistance of polarized H4-1 cells and a significant reduction in *L. monocytogenes* invasion in undifferentiated gut model cells. The latter characteristic was also shown by the *Lb. pentosus* S3T60C and *Lb. plantarum* S2T10D strains, whereas the increase of transepithelial resistance was shown by *Lb. plantarum* S1T10A, as well.

*In vivo* assays in mice and clinical trials are now needed for a further characterization of these probiotic candidates before their incorporation into novel functional foods.

## Supporting Information

Table S1 **Overall digestion survival (ODS), hydrophobicity (H), and autoaggregation (AA) of the 238** **LAB collected from green table olives.** Data are the means of three independent experiment and are represented in percentage (%). ODS is obtained by comparison of the initial lactobacilli counts at the start of the simulated digestion (T_0_) and those remaining at the end of the simulation (T_i_), according to the formula: T_i/_T_0_× 100. The H values are calculated using the formula: H = (1 − A_1_/A_0_)×100; where A_0_ represents the initial absorbance at 600 nm (A600) of the bacterial suspension in o-xylene/PBS solution, whereas the A_1_ represents the A600 measured for the aqueous phase after 1 h of incubation. The AA is an index of the bacterial cells capability to aggregate among them. The data are expressed using the formula 1 − (A_5/_A_0_)×100, where the A_0_ represents the A600 of a well resuspended bacterial culture, and A_5_ the A600 of the upper phase recovered from the same suspension after 5 h of incubation. Potential inhibition of *L. monocytogenes* growth due to acidification is reported in the last column.
(DOCX)

Table S2 **Time dependent TEER of polarized H4 monolayer exposed to the 17 probiotic candidates,** ***Lb. rhamnosus*** **GG (probiotic reference) and** ***L. monocytogenes*** **(negative control).** Bacterial effect on TEER dynamics was tested with 10^7^ CFU mL^−1^ of bacterial inoculum. Data (n = 3; ± SEM) are expressed as the ratio (%) of TEER at time t in relation to the initial value (t_0_). Different letters (a, b, c) indicate significant differences among the values at *P*<0.05 (ANOVA with Duncan’s test).
(DOCX)
