# Supplementary material for: In Vitro Selection and Characterization of New Probiotic Candidates from Table Olive Microbiota
Source: PLoS One. 2014 Apr 8;9(4):e94457. doi: 10.1371/journal.pone.0094457 (PMC3979845; doi:10.1371/journal.pone.0094457)
Supplement: Table S1 — Overall digestion survival (ODS), hydrophobicity (H), and autoaggregation (AA) of the 238 LAB collected from green table olives. Data are the means of three independent experiment and are represented in percentage (%). ODS is obtained by comparison of the initial lactobacilli counts at the start of the simulated digestion (T0) and those remaining at the end of the simulation (Ti), according to the formula: Ti/T0× 100. The H values are calculated using the formula: H = (1 − A1/A0)×100; where A0 represents the initial absorbance at 600 nm (A600) of the bacterial suspension in o-xylene/PBS solution, whereas the A1 represents the A600 measured for the aqueous phase after 1 h of incubation. The AA is an index of the bacterial cells capability to aggregate among them. The data are expressed using the formula 1 − (A5/A0)×100, where the A0 represents the A600 of a well resuspended bacterial culture, and A5 the A600 of the upper phase recovered from the same suspension after 5 h of incubation. Potential inhibition of L. monocytogenes growth due to acidification is reported in the last column. (DOCX) [file pone.0094457.s001.docx]

**Table S1.**

| **Strain code** | **Species** | **ODS (%)** | **H (%)** | **AA (%)** | **Inhibition of**  ***L. monocytogenes* due to acidification** |
| --- | --- | --- | --- | --- | --- |
| O1T90E | *Lb. plantarum* | 0.00677 | 42.9 | 54.6 | Inhibition |
| S11T3E | *Lb. plantarum* | 0.00492 | 42.6 | 39.3 | Inhibition |
| O2T60C | *Lb. plantarum* | 0.00397 | 39.4 | 41.7 | Inhibition |
| S1T30B | *Lb. plantarum* | 0.00268 | 45.2 | 58.2 | Inhibition |
| O1T90B | *Lb. plantarum* | 0.00267 | 40.3 | 47.0 | Inhibition |
| S1T90A | *Lb. plantarum* | 0.00205 | 40.8 | 19.6 |  |
| S1T15C | *Lb. plantarum* | 0.00151 | 27.6 | 14.8 |  |
| S1T3B | *Lb. plantarum* | 0.00142 | 46.4 | 48.0 |  |
| S1T90B | *Lb. plantarum* | 0.00142 | 4.7 | 26.0 |  |
| S1 T10A | *Lb. plantarum* | 0.00118 | 50.8 | 45.1 |  |
| O4T10E | *Lb. plantarum* | 0.00091 | 46.7 | 39.7 |  |
| O1T30E | *Lb. plantarum* | 0.00087 | 25.3 | 15.8 |  |
| S1T60D | *Lb. plantarum* | 0.00085 | 26.3 | 11.9 | Inhibition |
| S1T30E | *Lb. plantarum* | 0.00078 | 37.5 | 17.8 |  |
| O3T15B | *Lb. plantarum* | 0.00056 | 37.9 | 48.0 |  |
| O1T30A | *Lb. plantarum* | 0.00053 | 32.6 | 11.1 |  |
| S1T30A | *Lb. plantarum* | 0.00053 | 24.8 | 20.2 |  |
| S1T60B | *Lb. plantarum* | 0.00048 | 12.6 | 22.0 |  |
| O1T90A | *Lb. plantarum* | 0.00046 | 16.0 | 20.5 |  |
| S1T15D | *Lb. plantarum* | 0.00043 | 20.8 | 19.9 | Inhibition |
| O1T60E | *Lb. plantarum* | 0.00039 | 2.0 | 18.0 |  |
| O11T30D | *Lb. plantarum* | 0.00038 | 45.2 | 48.6 |  |
| O1T8A | *Lb. plantarum* | 0.00036 | 21.6 | 15.0 |  |
| O1T15B | *Lb. plantarum* | 0.00032 | 6.1 | 9.1 |  |
| FO50E | *Ln. mesenteroides* | 0.00028 | 30.3 | 45.6 |  |
| O1T15A | *Lb. plantarum* | 0.00027 | 23.7 | 19.5 |  |
| O1T60A | *Lb. plantarum* | 0.00027 | 30.5 | 12.8 |  |
| O1T90C | *Lb. plantarum* | 0.00024 | 68.1 | 40.0 |  |
| O1T8B | *Lb. plantarum* | 0.00023 | 42.0 | 13.1 |  |
| O1T3B | *Lb. plantarum* | 0.00021 | 13.3 | 26.8 | Inhibition |
| S1T10B | *Lb. plantarum* | 0.00021 | 23.7 | 19.2 |  |
| O1T60B | *Lb. plantarum* | 0.00020 | 23.4 | 13.5 |  |
| O2T60D | *Lb. plantarum* | 0.00016 | 43.6 | 52.2 |  |
| O1T8E | *Lb. plantarum* | 0.00014 | 15.7 | 32.5 | Inhibition |
| S1T90D | *Lb. plantarum* | 0.00014 | 31.0 | 16.7 |  |
| S1T30C | *Lb. plantarum* | 0.00012 | 22.7 | 16.5 |  |
| O1T60D | *Lb. plantarum* | 0.00009 | 2.5 | 16.6 |  |
| O1T90D | *Lb. plantarum* | 0.00009 | 11.4 | 20.0 |  |
| FS50Q | *Ln. mesenteroides* | 0.00007 | 48.8 | 44.5 |  |
| S2T10D | *Lb. plantarum* | 0.00005 | 54.8 | 46.1 |  |
| S3T60C | *Lb. pentosus* | 0.00004 | 43.5 | 49.4 |  |
| FO50M | *Ln. mesenteroides* | 0.00004 | 20.7 | 14.7 |  |
| S3T10B | *Lb. plantarum* | 0.00003 | 6.4 | 21.7 |  |
| S4T90D | *Lb. pentosus* | 0.00003 | 3.7 | 13.8 |  |
| FO30D | *Ln. mesenteroides* | 0.00002 | 34.8 | 18.5 |  |
| S3T60D | *Lb. pentosus* | 0.00002 | 13.6 | 17.6 |  |
| S4T30C | *Lb. plantarum* | 0.00002 | 55.9 | 42.4 |  |
| O1T10B | *Lb. plantarum* | 0.00002 | 30.1 | 28.1 |  |
| S1T10D | *Lb. plantarum* | 0.00002 | 20.6 | 20.3 |  |
| FO50L | *Ln. mesenteroides* | 0.00002 | 16.7 | 19.7 |  |
| FS50D | *Ln. mesenteroides* | 0.00002 | 8.7 | 14.7 |  |
| O4T15B | *Lb. plantarum* | 0.00001 | 8.7 | 23.4 | Inhibition |
| S3T30D | *Lb. plantarum* | 0.00001 | 39.0 | 23.5 |  |
| S3T8B | *Lb. plantarum* | 0.00001 | 14.5 | 19.3 |  |
| O11T60B | *Lb. plantarum* | 0.00001 | 34.1 | 22.4 |  |
| O4T30E | *Lb. plantarum* | / | 21.2 | 20.2 |  |
| O2T15C | *Lb. plantarum* | / | 38.9 | 17.6 |  |
| O4T30C | *Lb. plantarum* | / | 57.0 | 24.6 |  |
| S4T90A | *Lb. pentosus* | / | 22.2 | 14.2 |  |
| S3T10E | *Lb. plantarum* | / | 12.3 | 27.1 |  |
| S2T15E | *Lb. plantarum* | / | 5.0 | 18.2 |  |
| O2T60E | *Lb. plantarum* | / | 87.8 | 44.2 |  |
| O4T30A | *Lb. plantarum* | / | 4.4 | 17.4 |  |
| S1T15A | *Lb. plantarum* | / | 52.0 | 18.8 |  |
| S11T30D | *Lb. plantarum* | / | 49.4 | 21.9 |  |
| S3T30C | *Lb. plantarum* | / | 71.5 | 24.5 |  |
| S4T15E | *Lb. plantarum* | / | 13.7 | 26.9 |  |
| O4T90C | *Lb. plantarum* | / | 11.1 | 23.3 |  |
| S3T30A | *Lb. plantarum* | / | 10.0 | 23.9 |  |
| S11T15A | *Lb. plantarum* | / | 32.7 | 26.2 |  |
| S3T30E | *Lb. plantarum* | / | 17.4 | 23.4 |  |
| O11T30C | *Lb. plantarum* | / | 14.4 | 24.1 |  |
| O2T60A | *Lb. plantarum* | / | 66.3 | 25.1 |  |
| O3T60B | *Lb. plantarum* | / | 28.7 | 17.3 |  |
| S2T60A | *Lb. plantarum* | / | 13.8 | 13.0 |  |
| O2T60B | *Lb. plantarum* | / | 21.4 | 20.4 |  |
| O11T10C | *Lb. plantarum* | / | 22.5 | 24.3 |  |
| S11T10A | *Lb. plantarum* | / | 35.6 | 20.5 |  |
| S2T8E | *Lb. plantarum* | / | 3.3 | 21.1 |  |
| S11T30B | *Lb. plantarum* | / | 66.1 | 22.0 |  |
| S11T8E | *Lb. plantarum* | / | 1.7 | 26.9 |  |
| S11T30C | *Lb. plantarum* | / | 3.7 | 21.6 | Inhibition |
| S3T30B | *Lb. plantarum* | / | 23.2 | 35.7 |  |
| S3T8C | *Lb. plantarum* | / | 39.9 | 22.2 |  |
| O2T90E | *Lb. plantarum* | / | 10.9 | 22.1 |  |
| O2T30D | *Lb. plantarum* | / | 7.5 | 35.0 |  |
| S1T10E | *Lb. plantarum* | / | 16.1 | 21.1 |  |
| S4T15B | *Lb. plantarum* | / | 41.1 | 17.4 |  |
| O1T3C | *Lb. plantarum* | / | 21.2 | 24.1 |  |
| S2T3C | *Lb. plantarum* | / | 2.4 | 24.5 |  |
| S3T8D | *Lb. plantarum* | / | 10.3 | 12.5 |  |
| O3T30A | *Lb. plantarum* | / | 15.5 | 19.9 |  |
| S11T30E | *Lb. plantarum* | / | 20.7 | 19.0 |  |
| S3T3B | *Lb. plantarum* | / | 20.5 | 21.0 |  |
| O1T60C | *Lb. plantarum* | / | 36.2 | 32.1 |  |
| S3T90D | *Lb. plantarum* | / | 32.5 | 10.0 | Inhibition |
| O11T30B | *Lb. plantarum* | / | 16.2 | 18.9 |  |
| O4T30D | *Lb. plantarum* | / | 1.2 | 22.8 |  |
| FO50C | *Ln. mesenteroides* | / | 3.7 | 13.8 |  |
| O11T15D | *Lb. plantarum* | / | 5.2 | 24.2 |  |
| S1T10C | *Lb. plantarum* | / | 57.3 | 18.7 |  |
| S1T90E | *Lb. plantarum* | / | 30.1 | 10.2 |  |
| S2T15B | *Lb. plantarum* | / | 30.9 | 22.7 |  |
| S2T15A | *Lb. plantarum* | / | 10.6 | 22.4 |  |
| S11T3A | *Lb. plantarum* | / | 33.6 | 24.6 |  |
| S4T30A | *Lb. plantarum* | / | 20.6 | 42.2 |  |
| O1T8C | *Lb. plantarum* | / | 28.7 | 25.1 |  |
| S11T8B | *Lb. plantarum* | / | 18.8 | 21.7 |  |
| S1T3A | *Lb. plantarum* | / | 1.0 | 16.9 |  |
| O1T10E | *Lb. plantarum* | / | 4.3 | 24.1 |  |
| S11T30A | *Lb. plantarum* | / | 0.5 | 16.4 |  |
| S2T3B | *Lb. plantarum* | / | 32.4 | 21.9 |  |
| S2T8C | *Lb. plantarum* | / | 39.2 | 28.8 |  |
| S2T60D | *Lb. pentosus* | / | 6.2 | 19.8 |  |
| FS30P | *Ln. mesenteroides* | / | 16.7 | 20.5 |  |
| O1T10D | *Lb. plantarum* | / | 25.7 | 22.0 |  |
| S1T8A | *Lb. plantarum* | / | 27.5 | 26.9 |  |
| S11T3B | *Lb. plantarum* | / | 35.8 | 27.9 |  |
| S11T15D | *Lb. plantarum* | / | 22.8 | 45.8 |  |
| O1T15C | *Lb. plantarum* | / | 21.5 | 11.6 |  |
| S1T8C | *Lb. plantarum* | / | 2.1 | 30.4 |  |
| S2T10A | *Lb. plantarum* | / | 10.1 | 17.3 |  |
| S2T30B | *Lb. plantarum* | / | 97.7 | 18.2 |  |
| O3T30D | *Lb. plantarum* | / | 18.9 | 28.9 |  |
| S1T30D | *Lb. plantarum* | / | 26.7 | 17.1 |  |
| S3T15E | *Lb. plantarum* | / | 6.8 | 23.3 |  |
| S11T15C | *Lb. plantarum* | / | 2.4 | 25.4 |  |
| O2T8C | *Lb. plantarum* | / | 5.4 | 23.3 |  |
| FO30A | *Ln. mesenteroides* | / | 28.0 | 18.0 |  |
| FS 30 O | *Ln. mesenteroides* | / | 20.7 | 14.2 |  |
| S2T3A | *Lb. plantarum* | / | 21.1 | 20.7 |  |
| S3T15B | *Lb. plantarum* | / | 33.0 | 39.3 |  |
| O3T60A | *Lb. plantarum* | / | 19.6 | 22.4 |  |
| O11T30A | *Lb. plantarum* | / | 63.6 | 23.6 |  |
| S2T90A | *Lb. pentosus* | / | 48.0 | 33.3 |  |
| O1T3A | *Lb. plantarum* | / | 7.2 | 21.5 |  |
| S1T15E | *Lb. plantarum* | / | 52.5 | 24.9 |  |
| O11T90C | *Lb. plantarum* | / | 12.2 | 2.8 | Inhibition |
| O2T15B | *Lb. plantarum* | / | 10.1 | 22.6 |  |
| S2T3D | *Lb. plantarum* | / | 0.2 | 28.4 |  |
| O2T90D | *Lb. plantarum* | / | 44.8 | 13.2 |  |
| S1T8E | *Lb. plantarum* | / | 17.8 | 17.1 |  |
| S1T90C | *Lb. plantarum* | / | 30.5 | 20.2 |  |
| S3T15A | *Lb. plantarum* | / | 8.3 | 12.8 |  |
| S3T15C | *Lb. plantarum* | / | 5.6 | 20.8 |  |
| O4T15C | *Lb. plantarum* | / | 20.8 | 42.9 |  |
| S2T10E | *Lb. plantarum* | / | 32.9 | 21.4 |  |
| S4T30B | *Lb. plantarum* | / | 8.3 | 21.4 | Inhibition |
| S3T3A | *Lb. plantarum* | / | 12.1 | 24.5 |  |
| O4T30B | *Lb. plantarum* | / | 2.3 | 23.3 |  |
| FS90 P | *Ln. mesenteroides* | / | 20.7 | 13.8 |  |
| S3T8A | *Lb. plantarum* | / | 11.0 | 22.1 |  |
| O11T60E | *Lb. plantarum* | / | 2.7 | 22.7 |  |
| S4T10D | *Lb. plantarum* | / | 42.5 | 19.6 |  |
| S11T10E | *Lb. plantarum* | / | 0.5 | 25.1 |  |
| S3T10A | *Lb. plantarum* | / | 6.4 | 19.2 |  |
| O1T10A | *Lb. plantarum* | / | 29.6 | 21.2 |  |
| S1T15B | *Lb. plantarum* | / | 6.9 | 30.2 |  |
| S1T90B | *Lb. plantarum* | / | 31.3 | 25.1 |  |
| S11T90D | *Lb. pentosus* | / | 14.0 | 11.8 |  |
| O3T30E | *Lb. plantarum* | / | 17.3 | 21.9 |  |
| S3T8E | *Lb. plantarum* | / | 9.3 | 27.3 |  |
| S2T8B | *Lb. plantarum* | / | 6.0 | 22.2 |  |
| S11T10C | *Lb. plantarum* | / | 42.0 | 21.8 |  |
| S3T3E | *Lb. plantarum* | / | 8.1 | 23.6 |  |
| S4T30E | *Lb. plantarum* | / | 3.4 | 18.0 |  |
| O3T3E | *Lb. plantarum* | / | 2.0 | 22.9 |  |
| S2T30E | *Lb. plantarum* | / | 95.9 | 24.8 |  |
| O1T3E | *Lb. plantarum* | / | 31.0 | 26.9 |  |
| S1T60C | *Lb. plantarum* | / | 14.7 | 38.2 |  |
| FO30O | *Ln. mesenteroides* | / | 3.7 | 15.4 |  |
| S3T15D | *Lb. plantarum* | / | 21.6 | 13.5 |  |
| S2T10C | *Lb. plantarum* | / | 2.8 | 20.6 |  |
| S11T15E | *Lb. plantarum* | / | 19.7 | 21.2 |  |
| S11T90A | *Lb. plantarum* | / | 8.3 | 14.6 |  |
| O4T15A | *Lb. plantarum* | / | 20.7 | 23.2 | Inhibition |
| FO30P | *Ln. mesenteroides* | / | 30.3 | 9.1 |  |
| FS90I | *Ln. mesenteroides* | / | 30.3 | 13.8 |  |
| O2T3D | *Lb. plantarum* | / | 0.2 | 27.4 |  |
| O2T90A | *Lb. pentosus* | / | 48.7 | 16.9 |  |
| S4T15A | *Lb. plantarum* | / | 6.6 | 26.2 |  |
| S11T60B | *Lb. pentosus* | / | 58.5 | 11.5 |  |
| FO50O | *Ln. mesenteroides* | / | 20.7 | 14.5 | Inhibition |
| O11T8D | *Lb. plantarum* | / | 17.7 | 15.1 | Inhibition |
| S11T10B | *Lb. plantarum* | / | 12.2 | 22.6 | Inhibition |
| O11T8E | *Lb. plantarum* | / | 5.5 | 23.8 |  |
| O11T60C | *Lb. plantarum* | / | 43.3 | 7.2 |  |
| S11T90C | *Lb. pentosus* | / | 6.2 | 33.2 |  |
| S4T15C | *Lb. plantarum* | / | 14.0 | 14.9 |  |
| O3T90E | *Lb. plantarum* | / | 3.9 | 19.0 |  |
| S2T3E | *Lb. plantarum* | / | 4.6 | 27.4 |  |
| O11T90E | *Lb. plantarum* | / | 3.1 | 48.2 |  |
| O3T10A | *Lb. plantarum* | / | 22.7 | 16.7 |  |
| S11T3C | *Lb. plantarum* | / | 48.4 | 19.8 |  |
| S11T8D | *Lb. plantarum* | / | 19.3 | 24.7 | Inhibition |
| O2T90B | *Lb. plantarum* | / | 59.6 | 8.9 |  |
| O11T3A | *Lb. plantarum* | / | 6.3 | 21.2 |  |
| S4T8E | *Lb. plantarum* | / | 25.2 | 20.5 |  |
| S11T3D | *Lb. plantarum* | / | 0.5 | 25.7 |  |
| FS90 N | *Ln. mesenteroides* | / | 16.7 | 14.5 |  |
| S2T8A | *Lb. plantarum* | / | 7.0 | 27.3 |  |
| O11T3B | *Lb. plantarum* | / | 21.8 | 19.5 | Inhibition |
| S3T10D | *Lb. plantarum* | / | 29.2 | 19.1 |  |
| S4T8D | *Lb. plantarum* | / | 40.1 | 9.1 | Inhibition |
| S2T60B | *Lb. plantarum* | / | 7.5 | 8.0 |  |
| S3T3D | *Lb. plantarum* | / | 37.8 | 17.9 |  |
| S4T60B | *Lb. pentosus* | / | 57.4 | 18.5 |  |
| S11T8A | *Lb. plantarum* | / | 18.3 | 18.7 |  |
| S11T60A | *Lb. plantarum* | / | 11.8 | 15.9 | Inhibition |
| S3T60A | *Lb. plantarum* | / | 46.3 | 8.8 |  |
| O4T8B | *Lb. plantarum* | / | 10.1 | 17.3 |  |
| S4T90C | *Lb. pentosus* | / | 34.8 | 14.5 |  |
| S3T90B | *Lb. plantarum* | / | 27.5 | 18.4 |  |
| O2T15E | *Lb. plantarum* | / | 10.2 | 19.9 |  |
| S11T90E | *Lb. plantarum* | / | 30.6 | 14.2 |  |
| S4T90B | *Lb. pentosus* | / | 28.0 | 14.7 |  |
| O11T3C | *Lb. plantarum* | / | 34.3 | 18.9 |  |
| S11T10D | *Lb. plantarum* | / | 34.6 | 24.4 |  |
| O3T60C | *Lb. plantarum* | / | 58.4 | 11.6 |  |
| S3T90A | *Lb. plantarum* | / | 3.4 | 13.9 |  |
| S3T60B | *Lb. plantarum* | / | 33.2 | 16.6 | Inhibition |
| S4T90E | *Lb. pentosus* | / | 30.3 | 15.6 |  |
| O3T90C | *Lb. plantarum* | / | 42.9 | 12.3 |  |
| S11T8C | *Lb. plantarum* | / | 4.4 | 30.8 |  |
| O11T10E | *Lb. plantarum* | / | 0.7 | 2.1 |  |
| S3T60E | *Lb. pentosus* | / | 42.3 | 16.0 |  |
| S4T15D | *Lb. plantarum* | / | 19.8 | 41.5 |  |
| O1T10C | *Lb. plantarum* | / | 26.9 | 22.4 |  |
| O1T15D | *Lb. plantarum* | / | 27.6 | 26.0 |  |
| O1T30B | *Lb. plantarum* | / | 15.7 | 15.7 |  |
| O1T30C | *Lb. plantarum* | / | 34.5 | 14.0 |  |
| O1T30D | *Lb. plantarum* | / | 36.7 | 15.5 |  |
| S1T60E | *Lb. plantarum* | / | 34.5 | 16.2 |  |
| O2T15A | *Lb. plantarum* | / | 36.5 | 18.2 |  |
| S4T8C | *Lb. pentosus* | / | 20.7 | 15.4 |  |
| O3T30C | *Lb. plantarum* | / | 11.3 | 24.0 |  |
| S11T90B | *Lb. plantarum* | / | 13.0 | 18.7 |  |
| S4T10C | *Lb. plantarum* | / | 0.4 | 19.6 |  |

“/”: undetectable values (< 0.00001 %), in which the counts after the simulated digestion were < 10 CFU mL^-1^.
