# Supplementary material for: In Vitro Selection and Characterization of New Probiotic Candidates from Table Olive Microbiota
Source: PLoS One. 2014 Apr 8;9(4):e94457. doi: 10.1371/journal.pone.0094457 (PMC3979845; doi:10.1371/journal.pone.0094457)
Supplement: Table S2 — Time dependent TEER of polarized H4 monolayer exposed to the 17 probiotic candidates, Lb. rhamnosus GG (probiotic reference) and L. monocytogenes (negative control). Bacterial effect on TEER dynamics was tested with 107 CFU mL−1 of bacterial inoculum. Data (n = 3; ± SEM) are expressed as the ratio (%) of TEER at time t in relation to the initial value (t0). Different letters (a, b, c) indicate significant differences among the values at P<0.05 (ANOVA with Duncan’s test). (DOCX) [file pone.0094457.s002.docx]

**Table S2.**

|  |  | **Exposure time** | | | | | | | | | | | | | | | | | | | | | | | | | | | | |
| --- | --- | --- | --- | --- | --- | --- | --- | --- | --- | --- | --- | --- | --- | --- | --- | --- | --- | --- | --- | --- | --- | --- | --- | --- | --- | --- | --- | --- | --- | --- |
|  |  | 1 h | | | |  | 3 h | | | |  | 5 h | | | |  | 7 h | | | |  | 18 h | | | |  | 24 h | | | |
| Control |  | 91.9 | ± | 23.2 | **^a,b^** |  | 125.4 | ± | 25.3 | **^a,b^** |  | 124.7 | ± | 17.0 | **^a,b^** |  | 127.8 | ± | 14.4 | **^a,b^** |  | 94.8 | ± | 4.8 | **^b^** |  | 110.8 | ± | 13.3 | **^b^** |
| O1T90C |  | 100.7 | ± | 14.7 | **^a,b^** |  | 117.2 | ± | 27.3 | **^a,b^** |  | 114.5 | ± | 25.4 | **^a,b^** |  | 117.1 | ± | 26.5 | **^b^** |  | 76.4 | ± | 5.7 | **^b,c^** |  | 86.5 | ± | 13.3 | **^b,c^** |
| O11T30D |  | 95.8 | ± | 17.3 | **^a,b^** |  | 106.1 | ± | 16.4 | **^a,b^** |  | 101.8 | ± | 10.7 | **^a,b^** |  | 105.3 | ± | 9.5 | **^b^** |  | 76.9 | ± | 11.8 | **^b,c^** |  | 86.9 | ± | 12.3 | **^b,c^** |
| O1T90E |  | 106.8 | ± | 12.0 | **^a,b^** |  | 109.5 | ± | 9.6 | **^a,b^** |  | 102.3 | ± | 7.5 | **^a,b^** |  | 103.3 | ± | 3.4 | **^b^** |  | 70.2 | ± | 3.9 | **^b,c^** |  | 83.4 | ± | 7.5 | **^b,c^** |
| S1T3B |  | 116.8 | ± | 37.4 | **^a^** |  | 98.1 | ± | 25.5 | **^a,b^** |  | 96.1 | ± | 16.4 | **^a,b^** |  | 95.3 | ± | 18.9 | **^b,c^** |  | 75.7 | ± | 6.2 | **^b,c^** |  | 96.4 | ± | 9.1 | **^b^** |
| O1T90B |  | 117.4 | ± | 12.6 | **^a^** |  | 106.4 | ± | 18.3 | **^a,b^** |  | 107.6 | ± | 13.1 | **^a,b^** |  | 113.6 | ± | 16.0 | **^b^** |  | 90.2 | ± | 15.9 | **^b^** |  | 113.7 | ± | 18.5 | **^b^** |
| O2T60C |  | 93.5 | ± | 31.3 | **^a,b^** |  | 87.5 | ± | 23.5 | **^a,b^** |  | 84.9 | ± | 16.5 | **^c^** |  | 87.0 | ± | 15.8 | **^c^** |  | 95.8 | ± | 7.4 | **^b^** |  | 109.1 | ± | 9.6 | **^b^** |
| O4T10E |  | 101.3 | ± | 7.9 | **^a,b^** |  | 97.6 | ± | 7.1 | **^a,b^** |  | 98.0 | ± | 9.3 | **^a,b^** |  | 100.8 | ± | 8.5 | **^b^** |  | 100.9 | ± | 12.8 | **^a,b^** |  | 102.7 | ± | 6.6 | **^b^** |
| FS 50 Q |  | 100.5 | ± | 13.2 | **^a,b^** |  | 117.6 | ± | 16.8 | **^a,b^** |  | 124.5 | ± | 21.3 | **^a,b^** |  | 134.5 | ± | 26.4 | **^a^** |  | 108.4 | ± | 15.3 | **^a,b^** |  | 129.5 | ± | 18.2 | **^a,b^** |
| FO 50 E |  | 111.6 | ± | 8.5 | **^a,b^** |  | 130.9 | ± | 7.8 | **^a^** |  | 129.4 | ± | 2.5 | **^a^** |  | 131.0 | ± | 3.7 | **^a^** |  | 110.5 | ± | 5.5 | **^a,b^** |  | 132.4 | ± | 10.0 | **^a,b^** |
| S4T30C |  | 96.8 | ± | 23.8 | **^a,b^** |  | 107.0 | ± | 27.7 | **^a,b^** |  | 110.6 | ± | 26.5 | **^a,b^** |  | 114.2 | ± | 26.9 | **^b^** |  | 112.6 | ± | 30.7 | **^a,b^** |  | 138.1 | ± | 19.6 | **^a,b^** |
| S1T30B |  | 89.7 | ± | 6.2 | **^a,b^** |  | 97.8 | ± | 7.4 | **^a,b^** |  | 99.3 | ± | 8.0 | **^a,b^** |  | 105.4 | ± | 11.8 | **^b^** |  | 93.8 | ± | 12.2 | **^b^** |  | 110.3 | ± | 20.4 | **^b^** |
| S1T10A |  | 86.3 | ± | 7.8 | **^a,b^** |  | 91.3 | ± | 15.0 | **^a,b^** |  | 95.3 | ± | 11.2 | **^a,b^** |  | 100.0 | ± | 15.0 | **^b^** |  | 141.1 | ± | 12.0 | **^a^** |  | 164.4 | ± | 16.2 | **^a^** |
| O3T15B |  | 103.4 | ± | 14.2 | **^a,b^** |  | 106.8 | ± | 10.8 | **^a,b^** |  | 102.8 | ± | 9.1 | **^a,b^** |  | 106.7 | ± | 9.3 | **^b^** |  | 115.7 | ± | 14.4 | **^a,b^** |  | 127.3 | ± | 19.5 | **^a,b^** |
| O2T60D |  | 81.1 | ± | 12.5 | **^b^** |  | 94.9 | ± | 14.1 | **^a,b^** |  | 97.9 | ± | 14.8 | **^a,b^** |  | 101.0 | ± | 14.3 | **^b^** |  | 110.4 | ± | 22.7 | **^a,b^** |  | 125.5 | ± | 23.2 | **^a,b^** |
| S3T60C |  | 106.4 | ± | 23.8 | **^a,b^** |  | 110.8 | ± | 26.3 | **^a,b^** |  | 109.8 | ± | 28.2 | **^a,b^** |  | 113.2 | ± | 30.4 | **^b^** |  | 119.8 | ± | 18.8 | **^a,b^** |  | 133.8 | ± | 22.7 | **^a,b^** |
| S11T3E |  | 105.7 | ± | 11.3 | **^a,b^** |  | 116.2 | ± | 5.1 | **^a,b^** |  | 117.2 | ± | 3.0 | **^a,b^** |  | 123.2 | ± | 2.2 | **^b^** |  | 128.5 | ± | 9.5 | **^a^** |  | 139.5 | ± | 12.7 | **^a^** |
| S2T10D |  | 93.1 | ± | 5.4 | **^a,b^** |  | 104.7 | ± | 11.6 | **^a,b^** |  | 101.0 | ± | 10.8 | **^a,b^** |  | 104.4 | ± | 13.8 | **^b^** |  | 109.5 | ± | 15.9 | **^a,b^** |  | 114.6 | ± | 17.4 | **^b^** |
| LGG |  | 101.0 | ± | 7.0 | **^a,b^** |  | 116.8 | ± | 9.9 | **^a,b^** |  | 121.9 | ± | 14.0 | **^a,b^** |  | 127.5 | ± | 16.3 | **^a,b^** |  | 124.6 | ± | 27.0 | **^a,b^** |  | 130.8 | ± | 16.3 | **^a,b^** |
| Lmn |  | 103.1 | ± | 8.0 | **^a,b^** |  | 83.0 | ± | 2.4 | **^b^** |  | 65.3 | ± | 4.2 | **^c^** |  | 67.1 | ± | 4.7 | **^c^** |  | 56.4 | ± | 9.2 | **^c^** |  | 52.8 | ± | 6.5 | **^c^** |
